# Supplementary material for: NBM-BMX, an HDAC8 Inhibitor, Overcomes Temozolomide Resistance in Glioblastoma Multiforme by Downregulating the β-Catenin/c-Myc/SOX2 Pathway and Upregulating p53-Mediated MGMT Inhibition
Source: Int J Mol Sci. 2021 May 31;22(11):5907. doi: 10.3390/ijms22115907 (PMC8199487; doi:10.3390/ijms22115907)
Supplement: Supplementary file 1 [file ijms-22-05907-s001.zip › Supplementary Figure and Table.pdf]

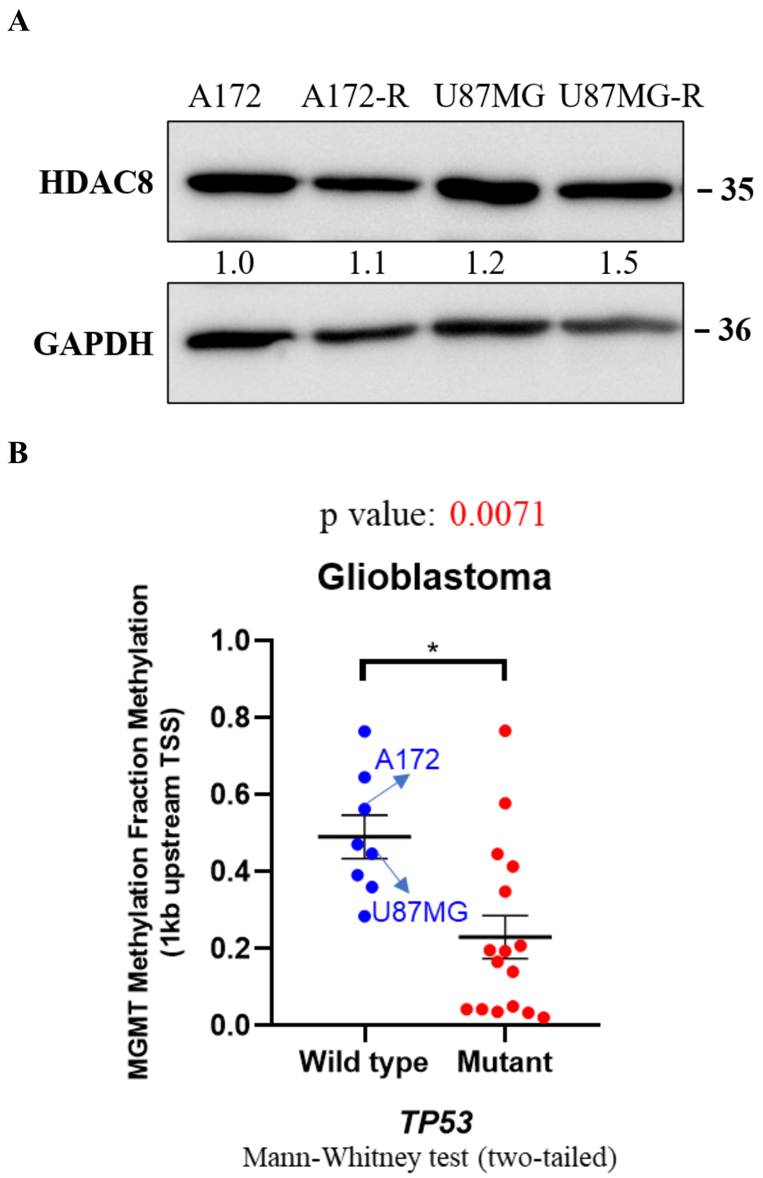

Figure S1. Genetic features of GBM cell lines. (A)The expression of HDAC8 from Abcam company in GBM cells by western blot. Supplementary Figure S2. BMX is a potent semi synthesized HDAC8 inhibitor. (B) The examination of MGMT methylation pairs GBM through big data analysis. The wide-type and mutatnt are labeled with blue and red, respectively (one-sided t test,* p<0.05).

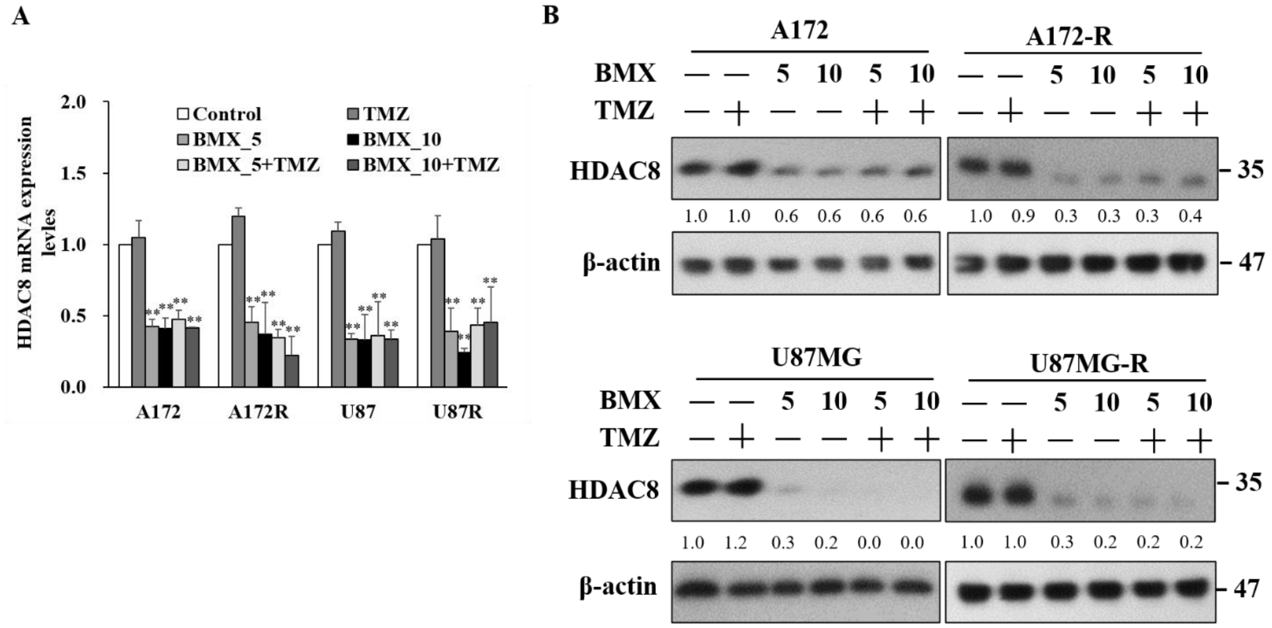

Figure S2. BMX is a potent semi synthesized HDAC8 inhibitor. (A) HDAC8 expression levels stimulated with different doses of BMX (0-10 μM) in the presence or absence of TMZ (50 μM) were determined using qRT-PCR assays. (B) HDAC8 expression levels stimulated with BMX (0-10 μM) with or without TMZ (50 μM) were determined using Western blotting.

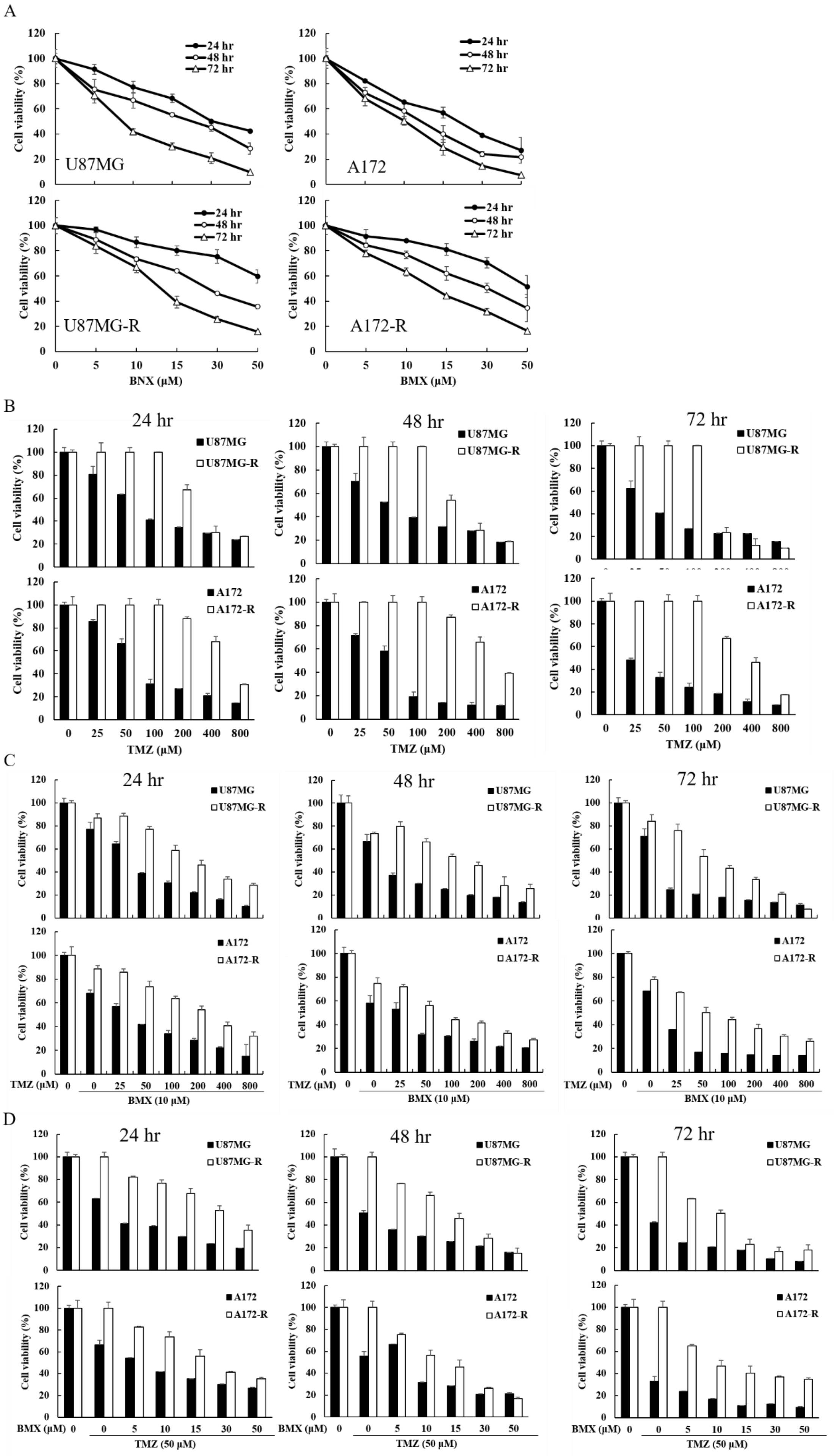
A
Cell viability (%)
24 hr
48 hr
72 hr
U87MG
A172
U87MG-R
A172-R
BNX (μM)
BMX (μM)
B
24 hr
48 hr
72 hr
U87MG
U87MG-R
A172
A172-R
TMZ (μM)
C
24 hr
48 hr
72 hr
TMZ (μM)
BMX (10 μM)
D
24 hr
48 hr
72 hr
BMX (μM)
TMZ (50 μM)

Figure S3. BMX and BMX with TMZ inhibits the growth and proliferation of U87MG, U87MG-R, A172 and A172-R**.** (A) Cell viability of GBM and GBM-R cell lines after treating with indicated concentrations of BMX (0.5, 10, 15, 30 or 50 µM) for 24, 48 and 72 hrs. (B) Cell viability of GBM and GBM-R cell lines after treating with the indicated concentrations of TMZ (0.25, 50, 100, 200, 400 or 800 µM) 24, 48 and 72 hrs. (C) Cell viability of GBM and GBM-R cells lines after treating with 10 µM BMX with or without TMZ at different concentrations (0.25, 50, 100, 200, 400 or 800 µM for 24, 48 and 72 hrs. (D) Cell viability of GBM and GBM-R cells lines after treating with 50µM TMZ with or without BMX at different concentrations (0.5, 10, 15, 30 or 50 µM) for 24, 48 and 72 hrs. (E) Cell viability of GBM-R cells lines after treating with 50 µM TMZ with or without 10 µM BMX for 24, 48 and 72 hrs.

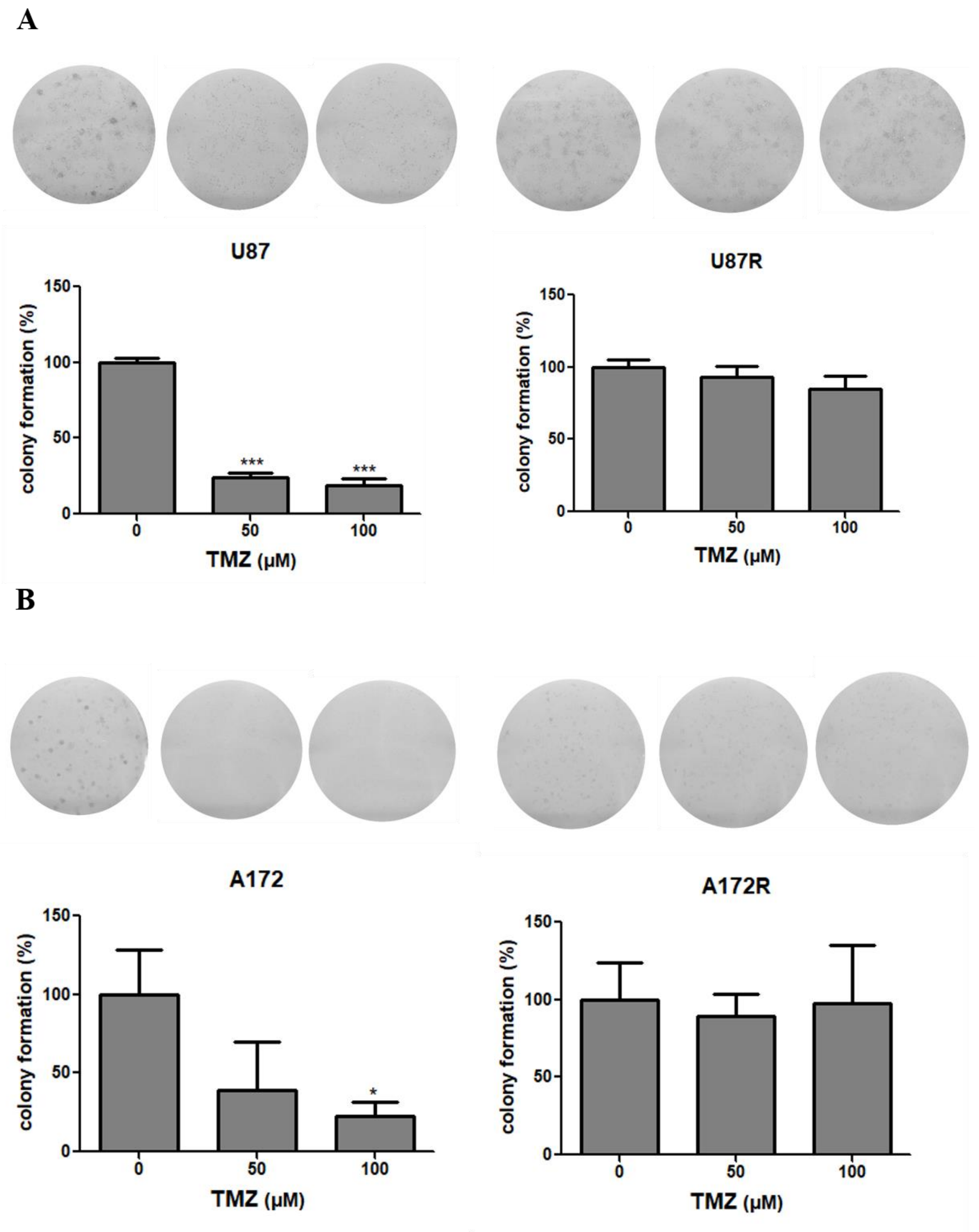

Figure S4. In vitro cytotoxicity of BMX in GBM cells. (A) U87 and U87R cells and (B) A172 and A172R cells were treated with TMZ (50 and 100 μM). This inhibitory effect of TMZ on GBM cells was determined using a clonogenic assay. *$p < 0.05$; **$p < 0.01$; ***$p < 0.001$.

Table S1. The drug and gene information in Glioblastoma.

Cell Line Name	cancer type	subtype	Temozolomide	MGMT			TP53			HDAC8		
			log2 fold change (Primary Screen)	Methylation Fraction (1kb upstream TSS)	Expression Public log2(TPM+1)	Relative Protein Expression	Mutation (one hot encoding)	Expression Public log2(TPM+1)	Relative Protein Expression	Mutation (one hot encoding)	Expression Public log2(TPM+1)	Relative Protein Expression
SF295	glioma	Glioblastoma	0.5225	0.19374	1.03562391	-2.139226981	1	5.819412726	2.393073817	0	4.502712486	#N/A
GB1	glioma	Glioblastoma	0.4427	0.41405	0.042644337	-3.805706295	1	7.565216347	2.73863664	0	4.874796966	#N/A
YKG1	glioma	Glioblastoma	0.3359	0.05019	4.146492307	#N/A	1	6.930737338	#N/A	0	5.270528942	#N/A
A172	glioma	Glioblastoma	0.3263	0.56288	0.084064265	-2.996286542	0	6.583308465	-1.299763044	0	4.631686366	1.192438806
KNS81	glioma	Glioblastoma	0.3098	0.39117	0.111031312	-2.080205897	0	6.587665021	-0.441223524	0	4.859969548	-1.749575686
YH13	glioma	Glioblastoma	0.3018	0.04314	3.601696516	#N/A	1	4.859472667	#N/A	0	5.073820233	#N/A
SNU466	glioma	Glioblastoma	0.2716	#N/A	3.980939266	#N/A	0	5.593054922	#N/A	0	4.496334513	#N/A
U87MG	glioma	Glioblastoma	0.1112	0.44678	0.070389328	-3.64685368	0	4.093391153	-1.870347741	0	4.618238656	-0.052702426
LN18	glioma	Glioblastoma	0.0981	#N/A	4.106850796	-0.031596888	1	5.890689878	2.254288844	0	5.314696526	-0.073179017
GAMG	glioma	Glioblastoma	0.0947	0.03358	0.555816155	-2.090445832	1	5.277984747	0.274042443	0	4.790772038	#N/A
T98G	glioma	Glioblastoma	0.0549	#N/A	3.360364277	#N/A	1	6.799216853	#N/A	0	4.676380255	#N/A
SNU489	glioma	Glioblastoma	-0.0105	0.03636	1.981852653	#N/A	1	4.685940148	#N/A	0	4.363871925	#N/A
SNU1105	glioma	Glioblastoma	**-0.0202**	0.19596	0.042644337	-1.942780912	1	5.516330387	-0.641586527	0	4.69432306	#N/A
DBTRG05MG	glioma	Glioblastoma	-0.0684	0.36067	0.028569152	#N/A	0	4.970853654	#N/A	0	4.818134761	#N/A
CAS1	glioma	Glioblastoma	-0.0712	0.20797	0.014355293	#N/A	1	6.077029314	#N/A	0	4.243364426	#N/A
KNS60	glioma	Glioblastoma	**-0.1272**	0.34877	2.799087306	#N/A	1	6.578334949	#N/A	0	4.329841177	#N/A
KNS42	glioma	Glioblastoma	-0.1520	#N/A	0.097610797	-2.561048733	1	5.231893162	-1.184272773	0	4.906409617	#N/A
GMS10	glioma	Glioblastoma	-0.2605	0.57795	0.070389328	#N/A	1	6.648753033	#N/A	0	5.146084939	#N/A
NMCG1	glioma	Glioblastoma	-0.2967	0.28477	0.124328135	#N/A	0	5.184280294	#N/A	0	4.258518925	#N/A
KALS1	glioma	Glioblastoma	-0.3007	0.14005	3.995484519	#N/A	1	6.468420402	#N/A	0	5.582254908	#N/A
LN229	glioma	Glioblastoma	-0.4896	0.76593	0.097610797	-2.376906118	1	5.590362488	-0.682367861	0	5.183089461	0.329211577
DKMG	glioma	Glioblastoma	-0.4971	0.76473	0.070389328	#N/A	0	5.472162804	#N/A	0	4.445594291	#N/A
SNU201	glioma	Glioblastoma	-0.5962	0.02159	0.970853654	#N/A	1	7.018923432	#N/A	0	4.756489607	#N/A
GOS3	glioma	Glioblastoma	-0.9443	0.64582	0.028569152	#N/A	0	6.235918488	#N/A	0	4.712045449	#N/A
GI1	glioma	Glioblastoma	-1.6327	0.44628	0.056583528	#N/A	1	5.975217457	#N/A	0	4.689858236	#N/A

high low

Methylation Fraction Gradient

high low

Expression Level Gradient

Table S2. CCLE p53 genetic features.

Tp53-WT		Tp53-mut	
SF126	KS1	LN229	YKG1
BECKER	MOGGUVW	SW1783	X8MGBA
GOS3	U87MG	TM31	MOGGCCM
KNS81	LN443	U138MG	
KG1C	LN235	SF295	
AM38	SF172	M059K	
H4	U343	GI1	
D283MED	U178	KNS60	
NMCG1	F5	KALS1	
YH13	SF767	CAS1	
DKMG	A1207	KNS42	
U118MG	LN319	SNU201	
GB1	LN382	SNU1105	
SNU489	LNZ308	SNU738	
SNU466	LN340	SNU626	
CCFSTTG1	CH157MN	DAOY	
D341MED	SF268	SW1088	
ONS76	SF539	HS683	
DBTRG05MG	SNB75	LN18	
A172		T98G	
X1321N1		GMS10	
SNB19		X42MGBA	
U251MG		GAMG	

Table S3. Experimental antibodies.

**Designation**	**Source or Reference**	**Identifiers**	**Additional Information**
GAPDH	Arigo, Hsinchu, Taiwan	ARG10112	1:5000
β-actin	Santa Cruz, CA, USA	sc-47778	1:2000
HDAC8	ABclonal, Woburn, MA, USA	A8865	1:1000
c-Myc	Cell Signaling Technology, Inc	#5605s	1:1000
cyclin D1	Cell Signaling Technology, Inc	#2978s	1:1000
cyclin B	Cell Signaling Technology, Inc	#12231s	1:1000
Caspase-3	(Pro and Active) Novus, USA	NB100-56708	1:1000
Bax	Cell Signaling Technology, Inc	#2772s	1:1000
p21	Cell Signaling Technology, Inc	#2947s	1:1000
Bcl-xL	Cell Signaling Technology, Inc	#2764s	1:1000
phospho-p53	p15;	#9284s	1:1000
puma	Cell Signaling Technology, Inc	#12450s	1:1000
MGMT	Cell Signaling Technology, Inc	#2739s	1:1000
p53	Santa Cruz, CA, USA	sc-126	1:1000
CD133	Cell Signaling Technology, Inc	#5860s	1:1000
CD44	Abcam, Cambridge, MA, USA	ab157107	1:1000
SOX-2	Cell Signaling Technology, Inc	ab97959	1:1000
